# Acute kidney injury is more common in men than women after accounting for socioeconomic status, ethnicity, alcohol intake and smoking history

**DOI:** 10.1186/s13293-021-00373-4

**Published:** 2021-04-08

**Authors:** Charalampos Loutradis, Luke Pickup, Jonathan P. Law, Indranil Dasgupta, Jonathan N. Townend, Paul Cockwell, Adnan Sharif, Pantelis Sarafidis, Charles J. Ferro

**Affiliations:** 1 Department of Renal Medicine, University Hospitals Birmingham NHS Foundation Trust, Birmingham, B15 2GW UK; 2 Department of Nephrology, Hippokration Hospital, Aristotle University of Thessaloniki, Thessaloniki, Greece; 3 Institute of Cardiovascular Sciences, College of Medical and Dental Sciences, Edgbaston, Birmingham, B15 2TT UK; 4 Warwick Medical School, University of Warwick, Coventry, CV4 7HL UK; 5 Department of Cardiology, University Hospitals Birmingham NHS Foundation Trust, Birmingham, B15 2GW UK

**Keywords:** Sex, Mortality, Acute kidney injury, Social status, Smoking, Alcohol

## Abstract

**Background:**

The association of several comorbidities, including diabetes mellitus, hypertension, cardiovascular disease, heart failure and chronic kidney or liver disease, with acute kidney injury (AKI) is well established. Evidence on the effect of sex and socioeconomic factors are scarce. This study was designed to examine the association of sex and socioeconomic factors with AKI and AKI-related mortality and further to evaluate the additional relationship with other possible risk factors for AKI occurrence.

**Methods:**

We included 3534 patients (1878 males with mean age 61.1 ± 17.7 and 1656 females 1656 with mean age 60.3 ± 20.0 years) admitted to Queen Elizabeth or Heartlands Hospitals, Birmingham, between October 2013 and January 2016. Patients were prospectively followed-up for a median 47.70 [IQR, 18.20] months. Study-endpoints were incidence of AKI, based on KDIGO-AKI Guidelines, and all-cause mortality. Data acquisition was automated, and information on mortality was collected from the Hospital Episode Statistics and Office of National Statistics. Socioeconomic status was evaluated with the Index of Multiple Deprivation (IMD).

**Results:**

Incidence of AKI was higher in men compared to women (11.3% vs 7.1%; *P* < 0.001). Model regression analysis revealed significant association of male sex with higher AKI risk (OR, 1.659; 95% CI, 1.311–2.099; *P* < 0.001); this association remained significant after adjustment for age, eGFR, IMD, smoking, alcohol consumption, ethnicity, existing comorbidities and treatment (OR, 1.599; 95% CI, 1.215–2.103; *P* = 0.001). All-cause mortality was higher in patients with compared to those without AKI. Males with AKI had higher mortality rates in the first 6-month and 1-year periods after the index AKI event. The association of male sex with mortality was independent of socioeconomic factors but was not statistically significant after adjustment for existing comorbidities.

**Conclusions:**

Men are at higher risk of AKI and this association is independent from existing risk factors for AKI. The association between male sex and AKI-related mortality was not independent from existing comorbidities. A better understanding of factors associated with AKI may help accurately identify high-risk patients.

**Supplementary Information:**

The online version contains supplementary material available at 10.1186/s13293-021-00373-4.

## Background

The reported incidence of acute kidney injury (AKI) in hospitalized patients is highly variable, depending on definitions and populations studied (5.4–18.3%), while incidence can increase to 40% in patients requiring admission to an intensive care unit [1, 2]. The most recent definition used for AKI is the one developed in the Kidney Disease: Improving Global Outcomes (KDIGO) AKI guidelines, which considers a diagnosis of AKI to be made when the serum creatinine (SCr) increases by 1.5–1.9 times or by ≥ 0.3 mg/dl (26.5 μmol/)l from previous known levels [3]. This variation is also affected by the reference value of SCr used for the diagnosis of AKI, i.e., whether the baseline SCr used is the admission SCr, or a preadmission SCr or an otherwise estimated SCr [4]. However, observational data suggest that occurrence of AKI is associated with a significantly increased mortality, longer admission and healthcare costs [4, 5]. Recent evidence from the UK Registry AKI report suggest that 71% (39% community and 32% hospital acquired) of people with an AKI episode had a 12-day hospital stay or more which results in an estimated cost of £620 million each year [6]. AKI occurrence also confers a higher risk for the development of chronic kidney disease (CKD) and end-stage kidney disease, which may contribute as an independent factor for adverse outcomes [7].

Several risk factors for AKI have been consistently identified, including older age, comorbidities such as cardiovascular disease, heart failure, CKD and diabetes, as well as severe infections and the use of nephrotoxic drugs [8]. Despite advances in knowledge on the pathophysiology of AKI, the mechanisms underlying the association between some of these conditions and AKI are not yet conclusively known [9]. Moreover, all of these established risk factors for AKI are more prevalent in men than women [10], and this may be influenced by socioeconomic status [11], alcohol intake [12], smoking status [13], and ethnicity [14]. Importantly, these factors commonly co-exist with each other and may present an additive effect on AKI occurrence [15].

In order to develop effective AKI prevention and management policies, a better understanding of the nature of these relationships is needed. However, the impact of the above sociodemographic factors on the incidence of AKI and subsequent outcomes have been less well studied. In particular, female sex is listed among the “shared susceptibility factors” that confers a higher risk of AKI according to the Kidney Disease Improving Global Outcomes (KDIGO) Clinical Practice Guideline for Acute Kidney Injury [3]. Furthermore, female sex is included as a risk factor in a number of situations including scoring models designed to predict the risk of AKI associated with the use of iodinated contrast media, aminoglycoside use, cardiac surgery and rhabdomyolysis [16–18]. These suggestions derive from low-quality observational studies in small populations; thus, the generalizability of such results is uncertain [19]. However, recent studies have reported a higher rate of AKI in men rather than women [20, 21]. To the best of our knowledge, no previous study has assessed whether an association between sex and the incidence of AKI or subsequent outcomes persist after adjusting for socioeconomic status, alcohol intake, smoking status, ethnicity and comorbidities that may mediate any association. Thus, this study was designed to examine whether sex is associated with the occurrence of AKI and with AKI-related mortality.

## Methods

### Study design and population

This is a secondary analysis of the ACQUATIK study, i.e., a prospective, multicentre, and observational study including patients admitted during the acute unselected medical takes at two large hospitals in Birmingham, i.e., the Queen Elizabeth Hospital and Birmingham Heartlands Hospital. The two hospitals treat over 2 million patients per year and perform over 2400 medical admissions on a monthly basis [22]. We enrolled consecutive patients, aged over 18 years, who were admitted during unselected medical takes (referred to the emergency room with a medical, rather than surgical problem, and not filtered by specialty) over a 27-month period (October 2013 to January 2016), with no a priori selection. Patients were approached to participate at any point during the index admission, but the reference date was the day of admission in all participants. Data was censored in September 2018 giving a maximum follow-up period of 59 months. Exclusion criteria included (i) renal replacement therapy (dialysis or transplantation), (ii) long-term follow-up by a renal team in secondary care, and (iii) attendance to renal clinic in secondary care during 12 months prior to study entry. All participants provided informed written consent and filled in a questionnaire assisted by a research nurse including demographic details and admissclinic in secondary careion medication for the evaluation of baseline characteristics. Patients were asked questions on self-reported ethnicity, awareness of any prior diagnosis of CKD, weekly alcohol unit intake, and smoking history. Power estimation was performed on the basis of the primary outcome of the ACQUATIK study i.e., the difference in readmissions between patients with and without CKD, as reported elsewhere [23]. The ACQUATIK study was approved by the National Research Ethics Service Committee West Midlands-South Birmingham Reference 13/WM/0317. The full rationale and design of the ACQUATIK study have been previously published [23].

### Data collection and definitions

Information on patient’s history and data from the index hospital admission and follow-up data were collected from the Hospital Episode Statistics (HES), which collates information on patients admitted to all NHS hospitals in England. Information for the haematology and biochemistry tests throughout the index admission were electronically collected from the individual hospital electronic health records (EHR). The HES also provides prognostic classification of existing comorbidities by evaluating the Charlson Comorbidity Index (CCI) [24]. Diagnosis of admission was categorized according to specialty or the nature of the disease (e.g., cardiology, gastroenterology, musculoskeletal/trauma, malignancy, neurology, renal, respiratory, infection, other or not specified) by two independent investigators. When two or more diagnoses were present, patients were categorized according to diagnosis associated with the presenting symptoms. Patients with infection or malignancy were categorized separately regardless of the affected system. All diagnoses categories with incidence ≤ 100 were combined in the other category. If diagnoses were categorized differently, this was resolved by discussion between the two investigators (CL and LP). When agreement could not be reached, a third investigator (CF) would adjudicate and make the final decision after reviewing the case record. Deaths during the study period, including date and cause of death, were derived through data linkage with the Office of National Statistics (ONS), which by law records information on all deaths in England. Alcohol consumption was defined as moderate (≤ 14 or ≤ 21 units/week) or increased (> 14 or > 21 units), based on the weekly intake for women and men accordingly [25]. Current smokers were defined as those who reported consumption of at least one cigarette per week and ex-smokers were those who had completely refrained from smoking at least 1 month prior to study entry. Ethnicity was classified into the following categories: White, Black, Asian for patients of Indo-Asian or Indian-Subcontinent descendancy and Other for patients with any mixed ethnicity background. The Index of Multiple Deprivation (IMD) 2015 was used to assess socioeconomic status. The IMD combines factors of housing, social and economic issues to give a single deprivation score for small areas or neighbourhoods in England. The weighted aggregation index of IMD is generated based on thirty-seven separate indicators, organized across seven distinct domains of deprivation (income, 22.5%; employment, 22.5%; health and disability, 13.5%; education, skills and training, 13.5%; barriers to housing and services, 9.3%; crime, 9.3%; and living environment, 9.3%) [26]. The IMD scores are divided into quantiles, 1 representing the most deprived and 5 representing the least deprived areas. In our study, patients with IMD 4 and 5 were categorized together, due to the small number of cases. AKI was defined as per KDIGO guidelines [an increase in serum creatinine by ≥ 0.3 mg/dL (≥ 26.5 μmol/l) within 48 h or an increase in serum creatinine of at least 1.5 times the baseline which is known or presumed to have occurred within the previous 7 days] [27]. Baseline SCr was defined as the admission SCr. The difference between the highest SCr value during each inpatient episode (excluding the admission SCr) and the admission SCr value was used to define the severity of the AKI [3]. Urine output criteria were not used since electronic records of urine output were often incomplete.

### Statistical analysis

Analyses were performed using SPSS 22.0 (SPSS Inc., Chicago, IL, USA). Values of *P* < 0.05 (two-tailed) were considered statistically significant in all comparisons. Continuous variables are expressed as mean ± standard deviation (SD) for normally distributed variables or median and interquartile range [IQR] for non-normally distributed variables and compared using the t-test or Mann-Whitney *U* test, accordingly. Categorical variables are expressed as absolute and relative frequencies and were compared using the Chi-squared test. All variables used in the analysis had < 5% of values missing and were therefore treated as missing completely at random with case-wise deletion. Proportional hazards assumption across groups was evaluated with log minus log survival curves. Kaplan-Meier survival curves were drawn to assess differences between male and female patients with and without AKI for time-to-event data and compared using the Log-rank test. The association of sex with AKI occurrence and mortality was evaluated with stepwise logistic or Cox regression modelled analysis (backwards method). Adjustments were performed for socioeconomic parameters, existing habits, comorbidities, laboratory results and medication intake that could possibly be associated with the outcome of interest and may confound its association with sex. Odds ratios (OR) and hazard ratios (HR) are presented with 95% confidence intervals (95% CI). A *P* value threshold of < 0.15 was selected in order to retain all potential risk factors and minimize the chance of type II errors. To address confounding by the between-group differences in baseline parameters, we estimated a propensity score for the diagnosis of admission, ethnicity, IMD, smoking habit, alcohol intake, baseline renal function, anaemia, BMI and existing comorbidities. Propensity score matching was implemented between male and female patients (1:1 ratio) using the nearest-neighbour strategy and a matching tolerance of 0.0001%.

## Results

### Baseline characteristics

**Fig. 1:**
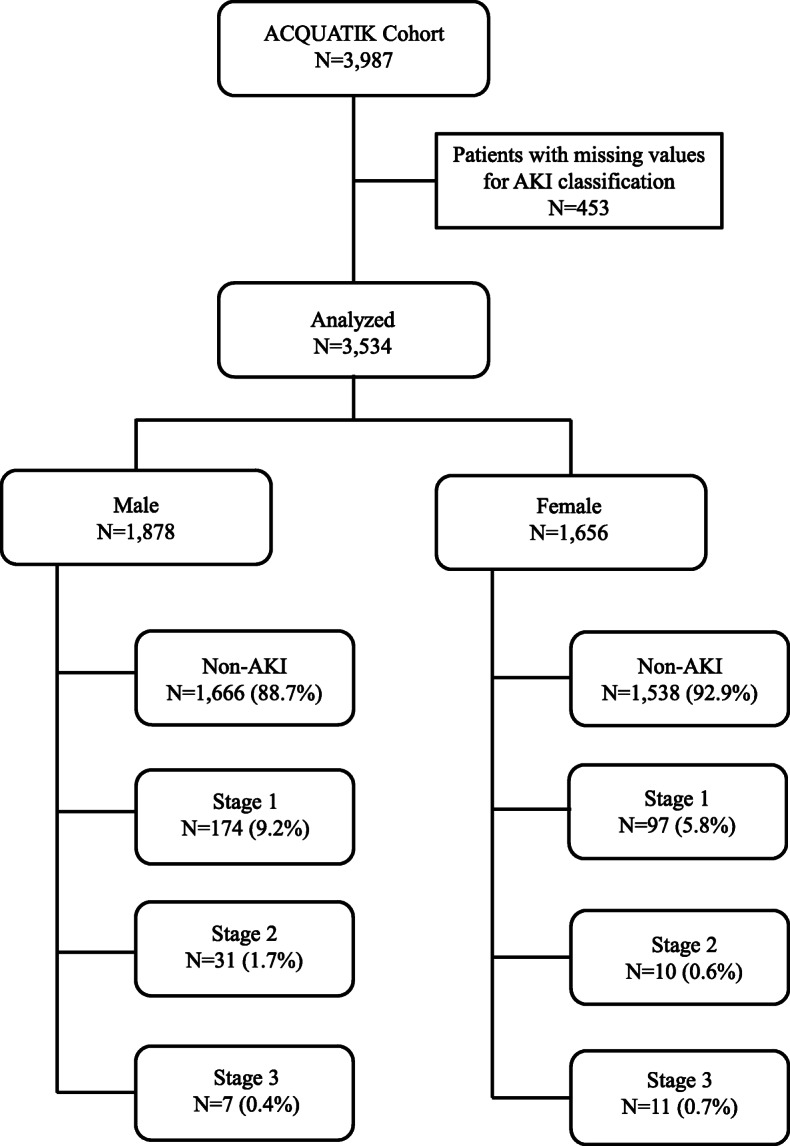
Patients’ enrolment and study flow diagram

As shown in Fig. 1, a total 3987 acute medical patients were recruited into the ACQUATIK study. We excluded 453 patients from this analysis because of missing values for AKI diagnosis. The remaining 3534 patients (1878 male vs 1656 female) were included and followed-up for a median of 47.70 [18.20] months. Baseline demographic, clinical and biochemical characteristics are presented in Table 1. The mean age of the population was 60.7 ± 18.8 years (male, 61.1 ± 17.7, vs female, 60.3 ± 20.0). No differences were evident between males and females in ethnicity and IMD. Women had significantly higher BMI compared to men [27.05 [7.50] vs 27.39 [9.90]; *P* = 0.03]. Prevalence of diabetes, hypertension, coronary heart disease, peripheral vascular disease, heart failure and malignancy were higher in male patients, while pulmonary disease was higher in females and previous stroke, renal and liver disease was similar between the two study groups. Male patients had higher CCI compared to females (1.22 ± 1.59 vs 1.05 ± 1.37; *P* = 0.021). Male patients had higher proportions of active (27.7% vs 24.2%) or previous smoking (41.4% vs 30.7%) and alcohol consumption (moderate, 29.1% vs 14.0%; high, 16.6% vs 7.1%). The number of patients receiving antihypertensive treatment was higher for males compared with females (48.7% vs 44.0%; *P* = 0.002). Estimated glomerular filtration rate (eGFR, 86.20 [35.94] vs 87.39 [37.32]; *P* = 0.08) was similar between the two groups. Distribution across the diagnosis of admission was different between males and females (Table 2).The baseline characteristics of the propensity-matched groups are presented in Supplementary table 1. The only parameters different between males and females were height, weight, smoking habit, and alcohol consumption, as well as e-GFR, creatinine, potassium, and haemoglobin levels, all being higher in males.

**Table 1:** Baseline characteristics in patients by sex and in the total population studied

Parameter	Total population	Male	Female	*P*
*N*	3534	1878	1656	-
Age (years)	60.7 ± 18.8	61.1 ± 17.8	60.3 ± 20.0	0.18
Ethnicity				
White (*n*, %)	3016 (85.3)	1589 (84.6)	1427 (86.2)	0.49
Black (*n*, %)	103 (2.9)	55 (2.9)	48 (2.9)	
Asian (*n*, %)	338 (9.6)	188 (10.0)	150 (9.1)	
Other (*n*, %)	77 (2.2)	46 (2.4)	31 (1.9)	
Index of multiple deprivation				
1	1571 (44.5%)	815 (43.4%)	756 (45.7%)	0.15
2	703 (19.9%)	366 (19.5%)	337 (20.4%)	
3	662 (18.7%)	363 (19.3%)	299 (18.1%)	
4–5	268 (7.6%)	157 (8.4%)	111 (6.7%)	
Height (m)	1.65 ± 0.12	1.71 ± 0.10	1.57 ± 0.10	**< 0.001**
Weight (kg)	77.29 ± 20.74	81.77 ± 19.28	72.18 ± 21.16	**< 0.001**
BMI (kg/m^2^)	27.16 [8.50]	27.05 [7.50]	27.39 [9.90]	**0.03**
Diabetes (*n*, %)	677 (19.2)	389 (20.7)	288 (17.4)	**0.01**
Hypertension (*n*, %)	1327 (37.5)	751 (40.0)	576 (34.8)	**0.001**
Coronary Heart Disease (*n*, %)	430 (12.2)	300 (16.0)	130 (7.9)	**< 0.001**
Stroke (*n*, %)	127 (3.6)	63 (3.4)	64 (3.9)	0.42
Peripheral Vascular Disease (*n*, %)	168 (4.8)	120 (6.4)	48 (2.9)	**< 0.001**
Heart Failure (*n*, %)	318 (9.0)	188 (10.0)	130 (7.9)	**0.02**
Renal Disease (*n*, %)	160 (4.5)	92 (4.9)	68 (4.1)	0.25
Liver Disease (*n*, %)	39 (1.1)	24 (1.3)	15 (0.9)	0.29
Pulmonary Disease (*n*, %)	860 (24.3)	380 (20.2)	480 (29.0)	**< 0.001**
Malignancy (*n*, %)	440 (12.5)	295 (15.7)	145 (8.8)	**< 0.001**
Charlson Comorbidity Index	1.14 ± 1.50	1.22 ± 1.59	1.05 ± 1.37	**0.021**
Smoking				
Current	921 (26.1%)	520 (27.7%)	401 (24.2%)	**< 0.001**
Ex-smoker	1286 (36.4%)	777 (41.4%)	509 (30.7%)	
Alcohol				
Moderate consumption (*n*, %)	779 (22.0)	547 (29.1)	232 (14.0)	**< 0.001**
Higher consumption (*n*, %)	430 (12.2)	312 (16.6)	118 (7.1)	
Antihypertensive drugs (*n*, %)	1642 (46.5)	914 (48.7)	728 (44.0)	**0.002**
ACEI (*n*, %)	698 (19.8)	428 (22.8)	270 (16.3)	**< 0.001**
ARB (*n*, %)	285 (8.1)	152 (8.1)	133 (8.0)	0.90
CCB (*n*, %)	595 (16.8)	344 (18.3)	251 (15.2)	**0.02**
Diuretics (*n*, %)	710 (20.1)	341 (18.2)	369 (22.3)	**0.002**
Beta-blockers (*n*, %)	569 (16.1)	353 (18.8)	216 (13.0)	**< 0.001**
Alpha1-blockers (*n*, %)	194 (5.5)	149 (7.9)	45 (2.7)	**< 0.001**
Statins (*n*, %)	430 (12.2)	249 (13.3)	181 (10.9)	0.06
Non-steroidal anti-inflammatory drugs (*n*, %)	109 (3.1)	54 (2.9)	55 (3.3)	0.47
Proton-pump inhibitors or histamine H2-receptor antagonists (*n*, %)	943 (26.7)	488 (26.0)	455 (27.5)	0.57
Haloperidol (*n*, %)	78 (2.2)	35 (1.9)	43 (2.6)	0.18
e-GFR (ml/min/1.73m^2^)	86.71 [36.59]	86.20 [35.94]	87.39 [37.32]	0.08
Creatinine (μmol/l)	74.00 [31.00]	83.00 [33.00]	64.50 [24.00]	**< 0.001**
Urea (mmol/l)	6.90 [4.20]	7.10 [4.00]	6.50 [4.40]	**< 0.001**
Sodium (mmol/l)	138.00 [4.00]	138.00 [4.00]	138.00 [4.00]	0.12
Potassium (mmol/l)	4.20 [0.60]	4.30 [0.60]	4.20 [0.60]	**< 0.001**
Haemoglobin (g/l)	125.00 [30.00]	129.00 [31.00]	120.00 [28.00]	**< 0.001**
Anaemia (*n*, %)	1730 (49.0)	937 (49.9)	793 (47.9)	0.267

*ACEI*, angiotensin converting enzyme inhibitors; *ARB*, angiotensin receptor blockers, *BMI*, body mass index; *CCB*, calcium channel blockers; *IMD*, index of multiple deprivation

Normally distributed variables are presented as mean ± standard deviation, non-normally distributed variables as median (interquartile range) and categorical variables as absolute frequency (proportion)

**Table 2:** Diagnosis of admission in patients by sex and in the total population studied

Parameter	Total population	Male	Female	*P*
*N*	3534	1878	1656	-
Cardiology (*n*, %)	707 (20.0)	446 (23.7)	261 (15.8)	**< 0.001**
Gastroenterology (*n*, %)	463 (13.1)	213 (11.3)	250 (15.1)	
Musculoskeletal/trauma (*n*, %)	407 (11.5)	174 (9.3)	233 (14.1)	
Malignancy (*n*, %)	275 (7.8)	179 (9.5)	96 (5.8)	
Neurology (*n*, %)	152 (4.3)	57 (3.0)	95 (5.7)	
Renal (*n*, %)	208 (5.9)	148 (7.9)	60 (3.6)	
Respiratory (*n*, %)	279 (7.9)	134 (7.1)	145 (8.8)	
Infection (*n*, %)	630 (17.8)	338 (18.0)	292 (17.6)	
Other (*n*, %)	294 (8.3)	124 (6.6)	170 (10.3)	
Not Specified (*n*, %)	119 (3.4)	65 (3.5)	54 (3.3)	

### Association of sex with risk of acute kidney injury

In total, 330 (9.3%) patients had AKI during admission. The incidence of AKI was higher in men compared to women (11.3% vs 7.1%; *P* < 0.001) with the majority of patients within each group having stage 1 AKI (males, 82.1%; females, 82.2%). In the propensity-matched cohort, incidence of AKI was again significantly higher in male compared to female patients (15.1% vs 10.2%; *P* = 0.003). In Table 3, we present the stepwise logistic analysis for the occurrence of AKI. Male sex was significantly associated with higher risk for AKI (OR, 1.659; 95% CI, 1.311–2.099; *P* < 0.001) in univariable analysis (model 1). The association between male sex and AKI persisted, with little modification, after adjustment for age, eGFR, IMD, smoking, alcohol consumption, ethnicity, existing comorbidities, treatment and laboratory variables (OR, 1.599; 95% CI, 1.215–2.103; *P* = 0.001). The comparisons in the propensity-matched groups were to the same direction indicating higher risk for AKI in male patients (Supplementary table 2).

**Table 3:** Stepwise logistic regression modelled analysis for the association of male sex with acute kidney injury

	Risk of AKI		Patients included in the model
	OR (95% CI)	***P***	
**Model 1**	1.659 (1.311–2.099)	**< 0.001**	3534
**Model 2**	1.670 (1.317–2.117)	**< 0.001**	3534
**Model 3**	1.640 (1.292–2.081)	**< 0.001**	3534
**Model 4**	1.628 (1.274–2.081)	**< 0.001**	3204
**Model 5**	1.573 (1.207–2.049)	**0.001**	2290
**Model 6**	1.599 (1.215–2.103)	**0.001**	2060

*AKI*, acute kidney injury; *BMI*, body mass index; *CI*, confidence intervals; *eGFR*, estimated glomerular filtration rate; *HR*, hazard ratio; *OR*, odds ratio; *Model 1*, unadjusted; *Model 2*, adjusted for age; *Model 3*, adjusted for age and eGFR; *Model 4*, adjusted for age, eGFR, indices of deprivation, smoking, alcohol consumption and race; Model 5, adjusted for age, eGFR, indices of deprivation, smoking, alcohol consumption, race, BMI, diabetes, hypertension, coronary heart disease, stroke, peripheral vascular disease, heart failure, renal disease, liver disease, pulmonary disease and malignancy; *Model 6*, adjusted for age, eGFR, indices of deprivation, smoking, alcohol consumption, race, BMI, diabetes, hypertension, coronary heart disease, stroke, peripheral vascular disease, heart failure, renal disease, liver disease, pulmonary disease, malignancy, antihypertensive medication intake, statin intake, sodium, potassium and haemoglobin levels

### Association of sex with mortality after acute kidney injury

The comparisons between patients with and without AKI for mortality rates within the male and female patient groups are presented in Table 4. In total, during the first 7 days after admission 9 patients died (6 males and 3 females) and 5 patients had AKI (2 males and 3 females). During the 30-day follow-up, all-cause mortality was higher in patients with than without AKI both in male (4.2% vs 1.8%; *P* = 0.04) and in female (0.8% vs 6.8%; *P* < 0.001) patients. The increased mortality in patients with AKI persisted in both males and females at 6 months, 1 year and 48 months with no differences between the sexes (Table 4). All-cause mortality was also higher in both males and females with AKI compared to those without AKI, with the exception of the 30-day follow-up period during which morality rate was numerically higher in male patients with AKI compared to those without AKI. During all follow-up periods, mortality was similar between males and females with AKI (Supplementary table 3).

**Table 4:** Comparisons of 30-day, 6-month, 1-year and 48-month all-cause mortality in male and female patients with and without AKI

Parameter	Male		*P*^#^	Female		*P*^#^	P*
	Without AKI	With AKI		Without AKI	With AKI		-
*N*	1666	212	-	1538	118	-	-
All-cause mortality in 30 days (*n*, %)	30 (1.8%)	9 (4.2%)	**0.04**	12 (0.8%)	8 (6.8%)	**< 0.001**	0.318
All-cause mortality in 6 months (*n*, %)	107 (6.4%)	42 (19.8%)	**< 0.001**	89 (5.8%)	18 (15.3%)	**< 0.001**	0.304
All-cause mortality in 1 year (*n*, %)	186 (11.2%)	56 (26.4%)	**< 0.001**	137 (8.9%)	25 (21.2%)	**< 0.001**	0.350
All-cause mortality in 48 months (*n*, %)	364 (21.8%)	83 (39.2%)	**< 0.001**	318 (20.7%)	56 (47.5%)	**< 0.001**	0.163

^#^Comparison between patients with and without AKI

*Comparison between male and female patients with AKI

*AKI*, acute kidney injury

**Fig. 2:**
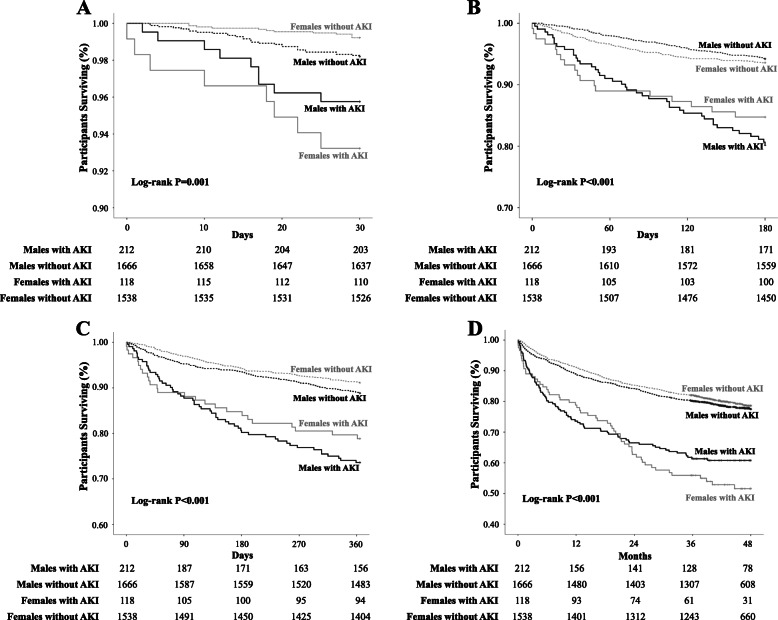
Kaplan-Meier curves in male and female patients with and without AKI during the **a** 30-day, **b** 6-month, **c** 1-year, and **d** 48-month follow-up periods

Figure 2 depicts the Kaplan-Meier curves and life tables in male and female patients with and without AKI over short-, mid- and long-term follow-up. Cumulative survival during the 30-day follow-up period was significantly different among the four groups; both males and females with AKI had lower survival compared to those without AKI (logrank-*P* = 0.001). Male patients with AKI had the lowest 6-month cumulative survival, followed by the females with AKI and the patients without AKI (logrank-*P* < 0.001). Results during the 1-year follow-up were to the same direction (logrank-*P* < 0.001). During the 48-month follow-up period, females had lower cumulative survival compared to males with AKI, while cumulative survival was higher in patients without AKI (logrank-*P* = 0.001). Comparisons for all-cause mortality in the propensity-matched male and female patients with and without AKI yielded significant differences between groups during all follow-up periods (Supplementary figure 1).

The stepwise Cox regression analysis for all-cause mortality after AKI is presented in Table 5. No significant association was evident between sex and 30-day all-cause mortality risk after AKI. Male sex was associated with increased risk for 6-month all-cause mortality after AKI (HR, 2.083; 95% CI, 0.199–3.619; *P* = 0.009). This association persisted after adjustment for age, eGFR, IMD, smoking status, alcohol consumption and ethnicity with little modification (models 2–4). However, the association between male sex and higher 6-month mortality after AKI was not significant after the inclusion of existing comorbidities in the model (HR, 1.521; 95% CI, 0.817–2.829; *P* = 0.19). During the 1-year follow-up period, male sex was associated with increased risk for all-cause mortality after AKI independently of age, eGFR, ethnicity, socioeconomic and lifestyle factors (HR. 1.352; 95% CI, 1.098–1.664; *P* = 0.005). In contrast, male patients presented increased risk for 48-month mortality after AKI only after fitting the model for age (HR. 1.442; 95% CI, 1.026–2.028; *P* = 0.04), but further adjustment rendered this association not statistically significant. The same analysis in propensity-matched males and females revealed similar risk for all-cause mortality after AKI in the two groups (Supplementary table 4).

**Table 5:** Stepwise Cox regression modelled analysis for the association of male sex with all-cause mortality after acute kidney injury occurrence during the 30-day and the 6-month follow-up periods in the total studied population

	30-day all-cause mortality after AKI		6-month all-cause mortality after AKI		1-year all-cause mortality after AKI		48-month all-cause mortality after AKI		Patients included in the model
	HR (95% CI)	***P***	HR (95% CI)	***P***	HR (95% CI)	***P***	HR (95% CI)	***P***	
**Model 1**	0.996, 0.384–2.582	0.99	2.083, 0.199–3.619	**0.009**	2.007, 1.252–3.216	**0.004**	1.334, 0.950–1.872	0.10	3534
**Model 2**	1.005, 0.387–2.606	0.99	2.159, 1.241–3.754	**0.006**	2.120, 1.321–3.402	**0.002**	1.442, 1.026–2.028	**0.04**	3534
**Model 3**	0.957, 0.369–2.482	0.93	2.003, 1.153–3.480	**0.01**	1.994, 1.243–3.200	**0.004**	1.351, 0.961–1.899	0.08	3534
**Model 4**	0.820, 0.307–2.194	0.69	1.996, 1.147–3.474	**0.02**	2.029, 1.250–3.293	**0.004**	1.257, 0.882–1.792	0.21	3204
**Model 5**	0.697, 0.242–2.006	0.50	1.521, 0.817–2.829	0.19	1.400, 0.821–2.389	0.22	0.978, 0.652–1.468	0.92	2290
**Model 6**	0.456, 0.151–1.376	0.16	1.405, 0.747–2.642	0.29	1.442, 0.806–2.578	0.22	1.032, 0.676–1.575	0.88	2060

*AKI*, acute kidney injury; *BMI*, body mass index; *CI*, confidence intervals; *eGFR*, estimated glomerular filtration rate; *HR*, hazard ratio; *OR*, odds ratio; *Model 1*, unadjusted; *Model 2*, adjusted for age; *Model 3*, adjusted for age and eGFR; *Model 4*, adjusted for age, eGFR, indices of deprivation, smoking, alcohol consumption and ethnicity; *Model 5*, adjusted for age, eGFR, indices of deprivation, smoking, alcohol consumption, ethnicity, BMI, diabetes, hypertension, coronary heart disease, stroke, peripheral vascular disease, heart failure, renal disease, liver disease, pulmonary disease and malignancy; *Model 6*, adjusted for age, eGFR, indices of deprivation, smoking, alcohol consumption, ethnicity, BMI, diabetes, hypertension, coronary heart disease, stroke, peripheral vascular disease, heart failure, renal disease, liver disease, pulmonary disease, malignancy, antihypertensive medication intake, statin intake, sodium, potassium and haemoglobin levels

## Discussion

This prospective cohort study was designed to evaluate the association between sex, AKI occurrence, and AKI-related mortality and to examine the magnitude of the association of several risk factors, including socioeconomic status on this association. Our results suggest that the AKI incidence was greater in males compared to females admitted during unselected medical takes. The association between AKI and male sex was stable after adjustments for a wide set of demographic, social, comorbidity, laboratory and medication parameters. Repeated analysis in propensity-matched groups confirmed the association between male sex and increased risk for AKI. All-cause mortality after AKI was higher in both men and women with AKI than in those without AKI. Men with AKI had higher mortality than women with AKI up to 12 months after hospital admission. The association between male sex and mortality was independent of social factors and habits but was rendered not statistically significant after adjustment for existing comorbidities.

Sex-gap is a common phenomenon in several diseases, such as hypertension, cardiovascular disease, heart failure and autoimmune diseases, with evidence favouring either male or female patients [28–30]. In general, men have higher mortality rates across all age groups, but women do worse in diseases severity, disability and other health outcomes [31]. Several mechanisms have been proposed to disproportionate outcomes in men and women, including hormonal and genetic differences, social factors, existing comorbidities and habits, work stress or hostility, exercise and access to medical care [32]. Regarding AKI, evidence from most, but not all studies suggest a protective effect of female sex on the development of AKI after ischemia-reperfusion injury using animal models, possibly due to the suppression of enhanced renal sympathetic nerve activity during renal ischemia and the reduction of post-ischemic glomerular endothelial hyperpermeability induced by oestrogens production [33–36]. Results from previous animal and human studies suggested that lower expression of mRNA and protein epidermal growth factor receptor in the glomeruli and kidney tubules, as well as sex differences in mitochondrial respiration, biogenesis, and dynamics may result in increased result in susceptibility of females to renal dysfunction [37].

Importantly, the KDIGO guidelines list female sex as a susceptibility factor, while several clinical scoring systems associate female sex with higher risk of AKI [3, 16–18]. These suggestions derive mainly from earlier small, low-quality observational studies; thus, the generalizability of such results and from one particular setting to the other is uncertain [19]. In contrast, results from later meta-analyses indicate that female sex may be associated with a lower incidence of AKI. In a meta-analysis by Grams et al., including > 1 million patients, male sex was associated with higher risk of AKI over a 4-year follow-up period, regardless of the eGFR or the albuminuria levels at baseline [38]. Results from another meta-analysis, including 28 studies, suggested that men had significantly higher risk for hospital-associated AKI compared to women (OR, 1.23; 95% CI, 1.11–1.36) [39]. Similarly, Neugarten et al. showed that male sex was again associated with higher risk for AKI development requiring renal replacement treatment compared to female sex (OR, 2.19; 95% CI, 2.15–2.22; *P* < 0.0001) in their meta-analysis [40]. Our study confirms these results by showing that men are at increased risk for AKI occurrence independently of several possible confounders or established AKI risk factors. Moreover, our study expands previous knowledge by using laboratory data for the diagnosis of AKI, rather that administrative codes, which present lower sensitivity compared with the current KDIGO consensus definition [41].

Evidence from the literature suggest that socioeconomic status is strongly associated with CKD, but the mechanism through which low-income associates with renal dysfunction is unclear. Preliminary data suggest that impaired access to health care may play a significant role [42]. An observational study using data from general practices in the UK, covering 7% of the UK population, found little evidence for an association between lower socioeconomic status (indicated by area deprivation) and risk of AKI in older patients who had diabetes [43]. However, recent studies using data from the Welsh national electronic AKI reporting system showed that lower socioeconomic status was associated with higher incidence of AKI and age-adjusted mortality following AKI, although incorporation of comorbidities was limited [44, 45]. A more recent study linking primary care data and hospital laboratory data in 2 large English hospitals found that both the risk of AKI, and mortality after AKI were both associated with social deprivation [46]. Unfortunately, these studies did not examine the relationship between AKI and other demographic factors known to be associated with social deprivation including ethnicity, smoking and alcohol consumption [38, 47]. Our study expands the existing knowledge by showing that the socioeconomic status, evaluated with IMD, does not affect the association between sex and AKI development.

Previous data on the association between smoking, alcohol consumption, ethnicity and sex with AKI occurrence are scarce. This mainly comes from the fact that discrimination among these factors is usually very difficult. Observational data suggest that smoking habit and increased alcohol consumption is more common in males or most deprived populations or specific ethnicities [47–49]. In contrast, several studies have shown that alcohol abuse is strongly associated with higher cardiovascular risk [50]. This association is superimposed upon the fact that patients in most deprived areas present higher prevalence of established risk factors for AKI, such as hypertension, diabetes and obesity [51]. An observational study, including 10,588 patients, showed that black ethnicity was associated with 30% higher risk for AKI and this association remained significant after adjustment for several demographic, cardiovascular risk factors, kidney markers and time-varying number of hospitalizations (HR. 1.20; 95% CI, 1.01–1.43; *P* = 0.04) but was attenuated after additional adjustment for differences in insurance (HR. 1.1; 95% CI, 0.91–1.34; *P* = 0.30) or income (HR. 1.09; 95% CI, 0.89–1.32; *P* = 0.40) [52]. The results of our study help to address these issues by showing that the risk for AKI occurrence was not affected after adjusting for smoking habit, alcohol consumption, ethnicity and socioeconomic status.

This study has several strengths. We included a large, ethnically diverse population and patients were followed-up for a median of 4 years. We carefully chose to record data on specific factors related with social status and habits, which could possibly confound the observed differences between males and females. We followed a fully automated approach to acquire patients’ data and information on prespecified outcomes using the HES and ONS systems, which resulted in very complete data collection with very few missing values. We also used the IMD score to subjectively acquire information on the socioeconomic status of the patients included. Although propensity score methods may not fully eliminate confounding variables, repeated analysis in propensity-matched groups further support the association between male sex and increased risk for AKI. However, there are also limitations that need to be acknowledged. We used the admission SCr as the baseline SCr for both the diagnosis and the classification of severity of AKI. However, it is possible that the AKI was already established prior to admission and this would potentially reduce both the reported incidence and severity of AKI. The optimum way of diagnosing AKI is still unclear and other methods of establishing the baseline renal function are currently being investigated [53, 54]. We included an unselected population, which may have resulted in significant differences in several parameters between male and female patients and the inclusion of mainly white and most deprived patients. Although we have performed adjustment for several possible confounders through multivariable analyses and we also repeated the analysis in propensity-matched groups, this study is still observational in nature and the differences in AKI rates between men and women may still be explained by residual confounding. Information on alcohol intake and smoking habit were voluntarily provided by study participants and therefore could not be independently verified. Moreover, data on medications intake were recorded at study entry and the additional effect of nephrotoxic drugs intake on AKI occurrence during admission cannot be evaluated. Menstruation history was not recorded in our study and possible effect on our results cannot be excluded but it may be only minimal given that 30.4% of the women included were aged < 50 years. Although we included parameters with < 5% missing values in the analysis, the listwise deletion of patients with at least one missing value in the parameters included in the regression analyses has reduced the sample sizes in models 5 and 6. Consistent with most of the published literature on AKI, another limitation of this study may be that urine volume changes were not recorded and thus were not included in the diagnosis of AKI. Using reductions in urine volumes, in addition to serum creatinine to diagnose AKI might have increased the reported incidence of AKI although we cannot be certain of this. Moreover, possible between-group differences in the association between oliguria occurrence and outcomes cannot be excluded. Although we recorded information on many factors associated with AKI, the acquisition of data on other established risk factors, such as albuminuria, was not feasible.

### Perspectives and significance

To the best of knowledge, this is the first study evaluating the association of several sociodemographic factors on the association of sex with AKI and subsequent outcomes from longitudinal data. We found that men are at a higher risk for AKI occurrence and AKI-related mortal events. Although numbers of patients with severe AKI were very small, our data suggested that women may present with more severe AKI (Stage 3), but this did not appear to confer higher mortality rates. This needs to be further explored in future studies. Importantly, the association between sex and AKI occurrence or mortality after AKI was not affected by the social status, ethnicity, smoking habit and alcohol consumption of the patients. However, differences in mortality may be attributed, to an extent, to the higher prevalence of existing comorbidities in males. These findings provide a better understanding of risk factors for AKI leading to adverse outcomes and provide additional evidence on the factors that should be considered when it comes to planning future studies on interventions to prevent AKI.

## Supplementary Information

**Additional file 1: Table S1.** Baseline characteristics in patients by sex and in the propensity-matched population sample. **Table** **S2.** Stepwise logistic regression modeled analysis for the association of male sex with acute kidney injury in the propensity matched population. **Table S3.** Comparisons of 30-day, 6-month, 1-year and 48-month all-cause mortality in propensity matched male and female patients with and without AKI. **Table S4.** Stepwise Cox regression modeled analysis for the association of male sex with all-cause mortality after acute kidney injury occurrence during the 30-day and the 6-month follow-up periods in the the propensity-matched population sample. **Figure S1.** Kaplan Meier curves in propensity-matched male and female patients with and without AKI during the (A) 30-day, (b) 6-month, (C) 1-year and (D) 48-month follow-up periods.

## Data Availability

Technical appendix, statistical code, and dataset available from the corresponding author. Informed consent for data sharing was not obtained.

## Abbreviations

ACEI: Angiotensin converting enzyme inhibitors
AKI: Acute kidney injury
ARB: Angiotensin receptor blockers
BMI: Body mass index
CCB: Calcium channel blockers
CI: Confidence intervals
CKD: Chronic kidney disease
eGFR: Estimated glomerular filtration rate
EHR: Electronic health records
HES: Hospital Episode Statistics
IMD: Index of Multiple Deprivation
IQR: Interquartile range
KDIGO: Kidney Disease Improving Global Outcomes
HR: Hazard ratio
OR: Odds ratio
SCr: Serum creatinine
SD: Standard deviation
