# Supplementary material for: Acute kidney injury is more common in men than women after accounting for socioeconomic status, ethnicity, alcohol intake and smoking history
Source: Biol Sex Differ. 2021 Apr 8;12:30. doi: 10.1186/s13293-021-00373-4 (PMC8034098; doi:10.1186/s13293-021-00373-4)

**TITLE PAGE**

**Title: Acute kidney injury is more common in men than women after accounting for socioeconomic status, ethnicity, alcohol intake and smoking history.**

**Running head:** Acute kidney injury and sex

**Authors:** Charalampos Loutradis MD MSc PhD,^1, 2^ Luke Pickup MBChB MRCP,^3, 4^ Jonathan P Law MBChB MRCP,^1, 3^ Indranil Dasgupta DM FRCP,^1,4^ Jonathan N Townend MD FRCP, ^3, 5^ Paul Cockwell PhD FRCP,^1^ Adnan Sharif MD FRCP,^1^ Pantelis Sarafidis MD MSc PhD,^2^ Charles J Ferro MD FRCP^1,3^

**Affiliations:** (1) Department of Renal Medicine, University Hospitals Birmingham NHS Foundation Trust, Birmingham, UK, B15 2GW; (2) Department of Nephrology, Hippokration Hospital, Aristotle University of Thessaloniki, Thessaloniki, Greece; (3) Institute of Cardiovascular Sciences, College of Medical and Dental Sciences, Edgbaston, Birmingham, UK, B15 2TT; (4) Warwick Medical School. University of Warwick, UK, CV4 7HL; (5) Department of Cardiology, University Hospitals Birmingham NHS Foundation Trust, Birmingham, UK, B15 2GW.

**Corresponding author:** Prof Charles J Ferro, Department of Renal Medicine, Queen Elizabeth Hospital, Birmingham B15 2WB, UK.

Email: [charles.ferro@uhb.nhs.uk](mailto:charles.ferro@uhb.nhs.uk); Tel: +44 121 3715839; Fax: +44 121 3715858

**Supplementary Table 1:** Baseline characteristics in patients by sex and in the propensity-matched population sample.

| Parameter | Total population | Male | Female | P |
| --- | --- | --- | --- | --- |
| N | 1168 | 584 | 584 | - |
| Age (years) | 61.28±18.79 | 61.10±17.92 | 61.46±19.65 | 0.741 |
| Ethnicity |  |  |  |  |
| White (n, %) | 1036 (88.7%) | 513 (87.8%) | 523 (89.6%) | 0.381 |
| Black (n, %) | 33 (2.8%) | 16 (2.7%) | 17 (2.9%) |  |
| Asian (n, %) | 78 (6.7%) | 46 (7.9%) | 32 (5.5%) |  |
| Other (n, %) | 21 (1.8%) | 9 (1.5%) | 12 (2.1%) |  |
| Index of multiple deprivation |  |  |  |  |
| 1 | 516 (44.2%) | 265 (45.4%) | 251 (43.0%) | 0.748 |
| 2 | 283 (24.2%) | 134 (22.9%) | 149 (25.5%) |  |
| 3 | 271 (23.2%) | 135 (23.1%) | 136 (23.3%) |  |
| 4-5 | 98 (8.4%) | 50 (8.6%) | 48 (8.2%) |  |
| Height (m) | 1.65±0.11 | 1.71±0.09 | 1.58±0.09 | **<0.001** |
| Weight (kg) | 77.23±21.22 | 82.62±20.54 | 71.84±20.51 | **<0.001** |
| BMI (kg/m^2^) | 26.99 [8.74] | 26.78 [7.93] | 27.10 [9.53] | 0.575 |
| Diabetes (n, %) | 221 (18.9%) | 107 (18.3%) | 114 (19.5%) | 0.601 |
| Hypertension (n, %) | 457 (39.1%) | 231 (39.6%) | 226 (38.7%) | 0.764 |
| Coronary Heart Disease (n, %) | 130 (11.1%) | 69 (11.8%) | 61 (10.4%) | 0.457 |
| Stroke (n, %) | 48 (4.1%) | 26 (4.5%) | 22 (3.8%) | 0.555 |
| Peripheral Vascular Disease (n, %) | 52 (4.5%) | 26 (4.5%) | 26 (4.5%) | 1.000 |
| Heart Failure (n, %) | 117 (10.0%) | 61 (10.4%) | 56 (9.6%) | 0.626 |
| Renal Disease (n, %) | 63 (5.4%) | 31 (5.3%) | 32 (5.5%) | 0.897 |
| Liver Disease (n, %) | 14 (1.2%) | 7 (1.2%) | 7 (1.2%) | 1.000 |
| Pulmonary Disease (n, %) | 298 (25.5%) | 148 (25.3%) | 150 (25.7%) | 0.893 |
| Malignancy (n, %) | 154 (13.2%) | 77 (13.2%) | 77 (13.2%) | 1.000 |
| Charlson Comorbidity Index | 1.18±1.55 | 1.17±1.55 | 1.18±1.56 | 0.931 |
| Smoking |  |  |  |  |
| Current | 319 (27.3%) | 140 (24.0%) | 179 (30.7%) | **0.005** |
| Ex-smoker | 427 (36.6%) | 238 (40.8%) | 189 (32.4%) |  |
| Alcohol |  |  |  |  |
| Moderate consumption (n, %) | 221 (18.9%) | 96 (16.4%) | 125 (21.4%) | **<0.001** |
| Higher consumption (n, %) | 168 (14.4%) | 109 (18.7%) | 59 (10.1%) |  |
| Antihypertensive drugs (n, %) | 537 (46.0%) | 277 (47.4%) | 260 (44.5%) | 0.318 |
| ACEI (n, %) | 229 (19.6%) | 126 (21.6%) | 103 (17.6%) | 0.090 |
| ARB (n, %) | 85 (7.3%) | 43 (7.4%) | 42 (7.2%) | 0.910 |
| CCB (n, %) | 195 (16.7%) | 100 (17.1%) | 95 (16.3%) | 0.695 |
| Diuretics (n, %) | 255 (21.8%) | 112 (20.7%) | 143 (26.5%) | **0.025** |
| Beta-blockers (n, %) | 176 (15.1%) | 103 (19.1%) | 73 (13.5%) | **0.014** |
| Alpha1-blockers (n, %) | 72 (6.2%) | 58 (9.9%) | 14 (2.4%) | **<0.001** |
| Statins (n, %) | 98 (8.4%) | 46 (8.5%) | 52 (9.6%) | 0.519 |
| Non-steroidal anti-inflammatory drugs (n, %) | 51 (4.4%) | 23 (3.9%) | 28 (4.8%) | 0.474 |
| Proton-pump inhibitors or histamine H2-receptor antagonists (n, %) | 294 (25.2%) | 151 (28.0%) | 143 (26.5%) | 0.870 |
| Haloperidol (n,%) | 30 (2.6%) | 14 (2.6%) | 16 (3.0%) | 0.563 |
| e-GFR (ml/min/1.73m^2^) | 85.23 [40.93] | 85.05 [38.34] | 85.28 [42.30] | **<0.001** |
| Creatinine (μmol/l) | 74.00 [33.00] | 83.00 [34.75] | 66.00 [28.00] | **<0.001** |
| Urea (mmol/l) | 8.20 [3.30] | 8.10 [3.13] | 8.20 [3.60] | 0.278 |
| Sodium (mmol/l) | 139.00 [5.00] | 138.00 [4.00] | 139.00 [5.00] | 0.430 |
| Potassium (mmol/l) | 4.30 [0.70] | 4.30 [0.70] | 4.20 [0.60] | **0.044** |
| Haemoglobin (g/l) | 127.00 [28.75] | 130.00 [29.00] | 124.00 [26.00] | **<0.001** |
| Anemia (n, %) | 528 (45.2%) | 280 (47.9%) | 248 (42.5%) | 0.068 |

Abbreviations: ACEI, angiotensin converting enzyme inhibitors; ARB, angiotensin receptor blockers, BMI, body mass index; CCB, Calcium channel blockers; IMD, index of multiple deprivation

Normally distributed variables are presented as mean±standard deviation, non-normally distributed variables as median (interquartile range) and categorical variables as absolute frequency (proportion)

**Supplementary Table 2:** Stepwise logistic regression modeled analysis for the association of male sex with acute kidney injury in the propensity matched population.

|  | **Risk of AKI** | | **Patients included in the Model** |
| --- | --- | --- | --- |
|  | **OR (95%CI)** | **p** |  |
| **Model 1** | 1.719 (1.191-2.480) | **0.004** | 1168 |
| **Model 2** | 1.743 (1.206-2.518) | **0.003** | 1168 |
| **Model 3** | 1.742 (1.204-2.519) | **0.003** | 1168 |
| **Model 4** | 1.755 (1.209-2.546) | **0.003** | 1168 |
| **Model 5** | 1.747 (1.201-2.541) | **0.004** | 1168 |
| **Model 6** | 1.787 (1.217-2.625) | **0.003** | 1056 |

Abbreviations: AKI, acute kidney injury; BMI, body mass index; CI, confidence intervals; eGFR, estimated glomerular filtration rate; HR, hazard ratio; OR, odds ratio

**Model 1:** Unadjusted; **Model 2:** Adjusted for age; **Model 3:** Adjusted for age and eGFR; **Model 4:** Adjusted for age, eGFR, indices of deprivation, smoking, alcohol consumption and race; **Model 5:** Adjusted for age, eGFR, indices of deprivation, smoking, alcohol consumption, race, BMI, diabetes, hypertension, coronary heart disease, stroke, peripheral vascular disease, heart failure, renal disease, liver disease, pulmonary disease and malignancy; **Model 6:** Adjusted for age, eGFR, indices of deprivation, smoking, alcohol consumption, race, BMI, diabetes, hypertension, coronary heart disease, stroke, peripheral vascular disease, heart failure, renal disease, liver disease, pulmonary disease, malignancy, antihypertensive medication intake, statin intake, sodium, potassium and hemoglobin levels.

**Supplementary Table 3:** Comparisons of 30-day, 6-month, 1-year and 48-month all-cause mortality in propensity matched male and female patients with and without AKI

| Parameter | Male | | P# | Female | | P# | | P* |
| --- | --- | --- | --- | --- | --- | --- | --- | --- |
|  | Without AKI | With AKI | - | Without AKI | With AKI | | - | - |
| N | 500 | 84 | - | 532 | 52 | | - | - |
| All-cause mortality in 30 days (n, %) | 12 (2.4%) | 4 (4.8%) | 0.220 | 4 (0.8%) | 6 (11.5%) | | **<0.001** | 0.181 |
| All-cause mortality in 6 months (n, %) | 36 (7.2%) | 13 (15.5%) | **0.011** | 41 (7.7%) | 12 (23.1%) | | **0.001** | 0.362 |
| All-cause mortality in 1 year (n, %) | 55 (11.0%) | 20 (23.8%) | **0.001** | 62 (11.7%) | 15 (28.8%) | | **<0.001** | 0.514 |
| All-cause mortality in 48 months (n, %) | 106 (21.2%) | 30 (35.7%) | **0.004** | 143 (26.9%) | 26 (50.0%) | | **<0.001** | 0.100 |

# Comparison between patients with and without AKI

* Comparison between male and female patients with AKI

Abbreviations: AKI, acute kidney injury

**Supplementary Table 4:** Stepwise Cox regression modeled analysis for the association of male sex with all-cause mortality after acute kidney injury occurrence during the 30-day and the 6-month follow-up periods in the the propensity-matched population sample.

|  | **30-day all-cause mortality after AKI** | | **6-month all-cause mortality after AKI** | | **1-year all-cause mortality after AKI** | | **48-month all-cause mortality after AKI** | | **Patients included in the model** |
| --- | --- | --- | --- | --- | --- | --- | --- | --- | --- |
|  | **HR (95%CI)** | **p** | **HR (95%CI)** | **p** | **HR (95%CI)** | **p** | **HR (95%CI)** | **p** |  |
| **Model 1** | 0.671 (0.189-2.377) | 0.536 | 1.088 (0.497-2.385) | 0.833 | 1.335 (0.684-2.608) | 0.397 | 1.139 (0.674-1.926) | 0.627 | 1168 |
| **Model 2** | 0.677 (0.191-2.402) | 0.546 | 1.111 (0.506-2.436) | 0.794 | 1.388 (0.710-2.715) | 0.338 | 1.139 (0.674-1.926) | 0.627 | 1168 |
| **Model 3** | 0.684 (0.193-2.423) | 0.556 | 1.115 (0.509-2.445) | 0.785 | 1.368 (0.701-2.673) | 0.359 | 1.163 (0.687-1.968) | 0.573 | 1168 |
| **Model 4** | 0.809 (0.225-2.906) | 0.745 | 1.168 (0.530-2.574) | 0.701 | 1.360 (0.696-2.657) | 0.369 | 1.140 (0.672-1.935) | 0.627 | 1168 |
| **Model 5** | 0.923 (0.219-3.894) | 0.913 | 1.175 (0.524-2.634) | 0.695 | 1.290 (0.657-2.533) | 0.460 | 1.088 (0.632-1.872) | 0.761 | 1168 |
| **Model 6** | 0.398 (0.056-2.817) | 0.356 | 1.099 (0.472-2.560) | 0.827 | 1.194 (0.603-2.366) | 0.611 | 0.981 (0.558-1.724) | 0.947 | 1056 |

Abbreviations: AKI, acute kidney injury; BMI, body mass index; CI, confidence intervals; eGFR, estimated glomerular filtration rate; HR, hazard ratio; OR, odds ratio

**Model 1:** Unadjusted; **Model 2:** Adjusted for age; **Model 3:** Adjusted for age and eGFR; **Model 4:** Adjusted for age, eGFR, indices of deprivation, smoking, alcohol consumption and ethnicity; **Model 5:** Adjusted for age, eGFR, indices of deprivation, smoking, alcohol consumption, ethnicity, BMI, diabetes, hypertension, coronary heart disease, stroke, peripheral vascular disease, heart failure, renal disease, liver disease, pulmonary disease and malignancy; **Model 6:** Adjusted for age, eGFR, indices of deprivation, smoking, alcohol consumption, ethnicity, BMI, diabetes, hypertension, coronary heart disease, stroke, peripheral vascular disease, heart failure, renal disease, liver disease, pulmonary disease, malignancy, antihypertensive medication intake, statin intake, sodium, potassium and hemoglobin levels.

**Supplementary Figure 1:** Kaplan Meier curves in propensity-matched male and female patients with and without AKI during the (A) 30-day, (b) 6-month, (C) 1-year and (D) 48-month follow-up periods**.**


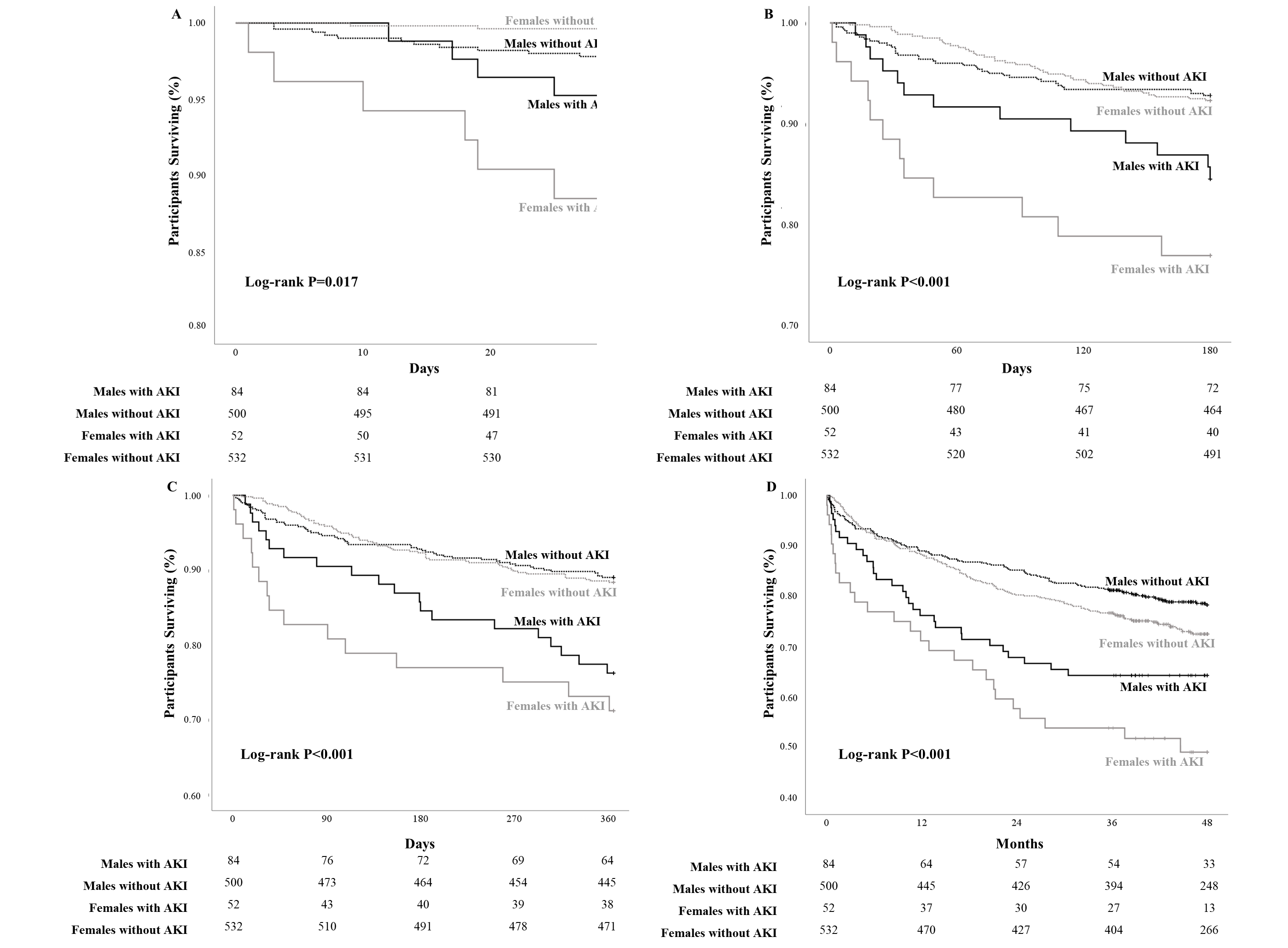

Supplement: Supplementary file 1 — Additional file 1: Table S1. Baseline characteristics in patients by sex and in the propensity-matched population sample. Table S2. Stepwise logistic regression modeled analysis for the association of male sex with acute kidney injury in the propensity matched population. Table S3. Comparisons of 30-day, 6-month, 1-year and 48-month all-cause mortality in propensity matched male and female patients with and without AKI. Table S4. Stepwise Cox regression modeled analysis for the association of male sex with all-cause mortality after acute kidney injury occurrence during the 30-day and the 6-month follow-up periods in the the propensity-matched population sample. Figure S1. Kaplan Meier curves in propensity-matched male and female patients with and without AKI during the (A) 30-day, (b) 6-month, (C) 1-year and (D) 48-month follow-up periods. [file 13293_2021_373_MOESM1_ESM.docx]
